# Oncostatin M orchestrates collective epithelial migration via HIF1A activation

**DOI:** 10.1038/s41540-026-00778-0

**Published:** 2026-07-06

**Authors:** Ian C. McLean, Sean M. Gross, Tiera A. Liby, Mark A. Dane, Daniel S. Derrick, Laura M. Heiser

**Affiliations:** 1 Department of Biomedical Engineering, Oregon Health & Science University, Portland, OR USA; 2 Capsida Biotherapeutics Inc., Thousand Oaks, CA USA; 3 Knight Cancer Institute, Oregon Health and Science University, Portland, OR USA

**Keywords:** Cancer, Cell biology, Computational biology and bioinformatics, Oncology

## Abstract

Extracellular signals strongly influence cell behavior, yet the mechanisms by which specific ligands mediate changes in phenotype remain unclear. The cytokine Oncostatin M (OSM) regulates homeostasis, wound healing, inflammation, and cancer progression. We previously found that OSM induces collective cell migration (CCM), a process whereby cells move as cohesive units while retaining cell-cell contacts, in MCF10A mammary epithelial cells. Here, we investigated how OSM drives CCM by comparing its effects with those elicited by epidermal growth factor (EGF) and interferon gamma (IFNG), defining ligand-specific phenotypes and molecular networks. Integrative transcriptomic and proteomic analyses identified hypoxia-inducible factor-1 (HIF1A) and signal transducer and activator of transcription 3 (STAT3) as central regulators of OSM responses. Functional validation revealed that HIF1A drives transcriptional programs associated with hypoxia, metabolic reprogramming, and immune pathways. Complement signaling emerged as a downstream effector of HIF1A, and its inhibition disrupted OSM-induced clustering and CCM. These findings establish a mechanistic link between OSM signaling, HIF1A activation, and CCM, demonstrating how cytokine-driven transcriptional reprogramming coordinates epithelial cell migration. Analysis of public breast cancer data indicate that this pathway is active in human tumors and may contribute to tissue remodeling, repair, and metastasis.

## Introduction

Collective cell migration (CCM) is a highly coordinated process in which groups of cells migrate together while maintaining cell-cell junctions and collective polarity^1^. Unlike single-cell migration, CCM depends on sustained intercellular communication and mechanical coupling to preserve collective integrity and directionality^2^. This coordinated behavior enables cells to respond to environmental cues during processes such as tissue morphogenesis, wound healing, and immune regulation^1,3–5^. CCM is driven by diverse signaling networks and cytoskeletal dynamics^2^, including maintenance of adherens junctions and coordinated cytoskeletal remodeling^6^. Cell-cell adhesion molecules such as E-cadherin maintain cohesion, while integrins mediate interactions with the extracellular matrix to provide traction and directional guidance^7^. Rho family GTPases regulate cytoskeletal rearrangements, enabling cells to generate the mechanical forces required for migration^8^. Extracellular cues, including growth factors and cytokines, modulate CCM by activating signaling pathways that regulate polarity, adhesion, and motility^9–12^. Despite its central role in multiple biological processes, the molecular mechanisms that coordinate CCM remain incompletely understood.

Beyond its roles in normal physiology, CCM has been implicated in cancer progression and metastasis. In breast cancer, tumor cells can adopt a collective migratory mode to invade surrounding tissues and disseminate to distant sites^13^. The maintenance of partial junctional integrity during CCM facilitates coordinated movement through the stroma^14,15^, while clusters of cancer cells exhibit improved survival during intravasation and extravasation, potentially due to retention of intercellular signaling and resistance to anoikis^16–19^. Collective migration in cancer is often associated with epithelial-to-mesenchymal transition (EMT), a state that enhances cellular plasticity and invasion while maintaining epithelial features^20^. Environmental cues such as interleukin-6 (IL-6) and hypoxia further promote collective invasion by enhancing tumor cell motility and stromal remodeling^21,22^. However, how specific inflammatory signals drive CCM through activation of specific molecular programs remain incompletely defined.

Among the diverse extracellular signals that regulate collective cell migration, Oncostatin M (OSM), an IL-6 family member, has recently been linked to the induction of CCM in epithelial cells^23^. OSM signals through a heterodimeric receptor composed of OSMRβ and gp130, activating downstream pathways including JAK-STAT, MAPK, and PI3K-AKT^24,25^. Treatment with OSM induces CCM in MCF10A cells, establishing this system as a tractable model for dissecting the signaling pathways underlying CCM^23^. MCF10A cells are a non-tumorigenic human mammary epithelial cell line that exhibits typical epithelial morphology, polarity, and responsiveness to microenvironmental cues, making them well suited for studying ligand-induced phenotypic transitions^23,26–32^. Our current study integrates live-cell imaging, transcriptomics, and functional assays to uncover the molecular programs driving CCM in MCF10A cells. We show that OSM activates a distinct transcriptional program in MCF10A cells, characterized by the upregulation of pathways associated with hypoxia and immune signaling. We identify hypoxia-inducible factor-1 (HIF1A) and signal transducer and activator of transcription 3 (STAT3) as central mediators of these responses and show that HIF1A contributes functionally to OSM-driven CCM. Finally, an analysis of publicly available breast cancer data demonstrates that OSM programs are associated with aggressive disease phenotypes.

## Results

### OSM induces collective migration in MCF10A epithelial cells

We previously reported that OSM induces changes in cell morphology and behavior, including collective cell migration (CCM)^23^. Here we dissected the molecular mechanisms underlying OSM-mediated CCM by comparing responses of OSM, epidermal growth factor (EGF), and interferon gamma (IFNG). OSM and IFNG both activate JAK-STAT signaling whereas EGF signals through receptor tyrosine kinase (RTK) pathways^12,24,33^. We chose IFNG as a comparator because it also activates JAK-STAT signaling but does not induce CCM. IFNG was delivered in combination with EGF because unlike OSM, IFNG alone does not support proliferation of MCF10A cells^23,34^.

**Fig. 1:**
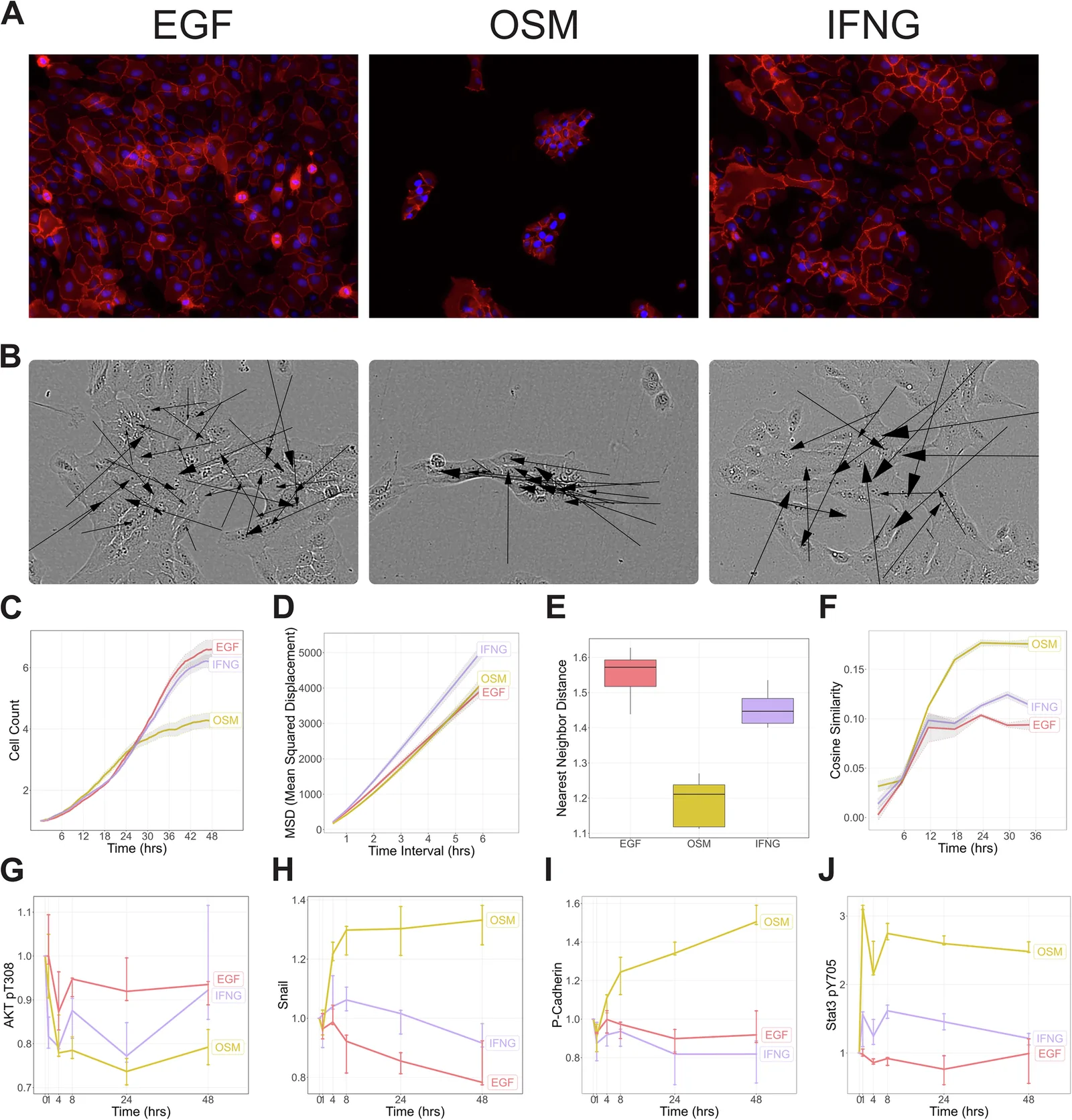
Treatment of MCF10A cells with OSM initiates cell clustering and collective cell migration. **A** MCF10A cells were treated with EGF, OSM, or IFNG for 48 hours, then stained for DAPI (blue) and β-Catenin (red). **B** Cell velocity vectors represent speed and direction of cell motility, derived from cell tracking data during 24–48 hours after ligand treatment. Arrow size is proportional to velocity. **C**–**F** Quantification of cell phenotype from live-cell imaging. Shaded region represents 95% confidence interval from 3 biological replicates (*n* = 3). Boxplots in (**E**) depict interquartile range of nearest neighbor distance calculated for each cell. **G**–**J** Line plots show protein expression levels normalized to the T0 control for each treatment condition. Error bars represent the full range of measurements (*n* = 3).

We treated MCF10A cells with OSM, EGF, or EGF + IFNG for 48 hours, and assessed their phenotypic responses with live-cell imaging and immunofluorescent microscopy. Staining with β-catenin at 48H revealed that EGF induces cells to form the characteristic cobblestone pattern typical of MCF10A epithelial sheets, while OSM treatment induces compact clusters of cells (Fig. 1A)^29^. Live-cell imaging revealed that OSM-induced migration occurs as cohesive clusters, a hallmark of CCM (Fig. 1B). This behavior was absent in both EGF and IFNG conditions.

We quantified several metrics to capture key features of the behaviors observed using live-cell imaging data: cell count to estimate proliferation, mean squared displacement (MSD) to estimate motility, nearest neighbor distance to assess cell clustering, and cosine similarity of displacement vectors to assess collective migration (Fig. 1C–F). Cell count was similar across conditions indicating maintenance of cell proliferation in all treatments (Fig. 1C, Supplementary Fig. 1A). MSD metrics revealed that IFNG treatment significantly increased motility of individual cells (*p* = 0.002), whereas OSM had no effect (Fig. 1D, Supplementary Fig. 1A). OSM significantly decreased nearest neighbor distance, indicating strong cell clustering (−22.3%, *p* < 0.001), while IFNG showed only a modest reduction (−6.9%, *p* = .023) (Fig. 1E, Supplementary Fig. 1A). Cosine similarity, a measure of coordinated movement, was strongly elevated in OSM-treated cells (*p* < 0.001) (Fig. 1F).

To identify molecular correlates of these phenotypic responses, we analyzed protein expression measured in publicly available reverse phase protein array (RPPA) data^23^. EGF and IFNG + EGF induced stronger AKT activation than OSM, consistent with their more proliferative phenotypes (Fig. 1G). RPPA further revealed that OSM uniquely upregulated Snail, an EMT-associated transcription factor (Fig. 1H). OSM also uniquely upregulated P-cadherin and modulated other junctional proteins (Connexin-43, β-catenin, Merlin), consistent with increased cell-cell adhesion and clustering (Fig. 1I). OSM and IFNG both activated STAT3 phosphorylation, though OSM induced a stronger response (Fig. 1J). Despite this shared JAK-STAT activation, only OSM promoted CCM, while IFNG enhanced motility without inducing clustering or Snail upregulation. This suggests that although both ligands engage JAK-STAT signaling, OSM promotes a distinct molecular program associated with collective migration. This observation motivated us to further explore the contrast between OSM and IFNG as a paradigm for dissecting the molecular mechanisms underlying CCM in epithelial cells.

### Comparative network analysis identifies OSM-specific molecular regulators

To dissect the molecular mechanisms driving OSM-induced CCM, we analyzed publicly available molecular profiling data from MCF10A cells treated with OSM or IFNG + EGF for 48 hours, including bulk RNA sequencing, RPPA, and cyclic immunofluorescence (CyCIF)^23,35,36^. Our goal was to identify molecular signatures uniquely activated by OSM as compared to IFNG, disentangling regulators of collective migration from general regulators of JAK-STAT signaling.

**Fig. 2:**
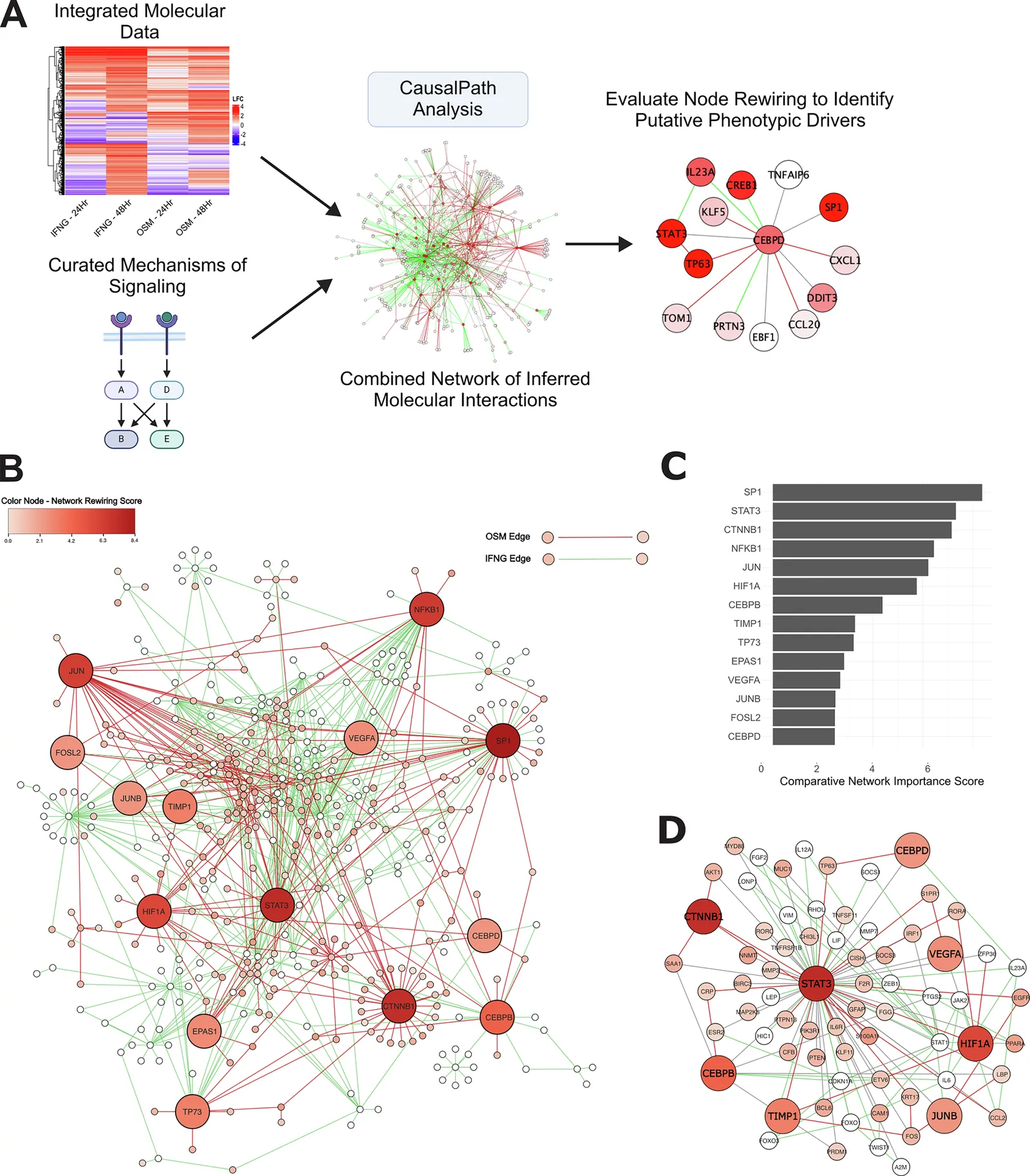
Comparative network analysis of omics data reveals putative drivers of OSM-induced CCM. **A** Workflow for the comparative network analysis to identify molecular subnetworks and nodes perturbed by OSM. Integrated molecular data collected from OSM and IFNG treated cells was analyzed using CausalPath. **B** Combined molecular network of OSM and IFNG activated nodes. The rewiring score between conditions is indicated by node color, and the top scoring nodes nominated for further investigation are indicated by increased size. **C** The top scoring nodes most rewired by OSM treatment, corresponding to the highlighted nodes in (B). **D** The majority (8/14) of top rewired nodes are centered around the STAT3 subnetwork.

We used CausalPath to infer network activity from transcriptional and proteomic data and visualized networks with Cytoscape^37,38^ (Fig. 2A, B Supplementary Fig. 2). We reasoned that molecular nodes showing the greatest differences in connectivity between OSM and IFNG conditions may act as key drivers of the OSM-induced CCM phenotype. To identify these candidates, we computed a rewiring score for each node, reflecting the number of edges that would need to be added or removed to shift its connectivity from one condition to the other^39^. In this context, edges represent regulatory or signaling interactions inferred by CausalPath, and the rewiring score therefore captures the extent to which a node’s local interaction network differs between conditions. Nodes with higher rewiring scores exhibit greater condition-specific changes in connectivity, suggesting a more prominent role in differentiating OSM versus IFNG-driven signaling. We weighted the rewiring metric by the log-fold change (LFC) induced by OSM for each node, prioritizing nodes that were both highly rewired and upregulated under OSM treatment.

STAT3 ranked second among nodes with the highest rewiring scores (Fig. 2C). This demonstrates the value of a network-based approach: while STAT3 is activated by both treatments, its functional connectivity is distinct, suggesting unique functional roles. Eight of the fourteen top-rewired nodes were centered around the STAT3 subnetwork, consistent with the pivotal role of STAT3 in cytokine signaling and regulation of cellular phenotypes (Fig. 2D).

Several transcription factors, including SP1, JUN family members, CTNNB1, TP73, and CEBP factors, were highly rewired, consistent with their roles in mediating cytokine signaling and regulation^40–45^. Nodes associated with hypoxia signaling, such as HIF1A, VEGFA, TIMP1, and EPAS1, also showed high rewiring. HIF1A, a master regulator of hypoxia, is particularly notable as prior studies have indicated it can be upregulated by OSM in normal and cancerous cellular contexts^46,47^. Hypoxia-related pathways contribute to modulating migratory phenotypes, including collective cell migration^20,22,48^. These findings suggest a mechanistic link between OSM signaling and HIF1A-associated regulation of migratory behavior.

### Functional validation reveals causal regulators of OSM-induced CCM

**Fig. 3:**
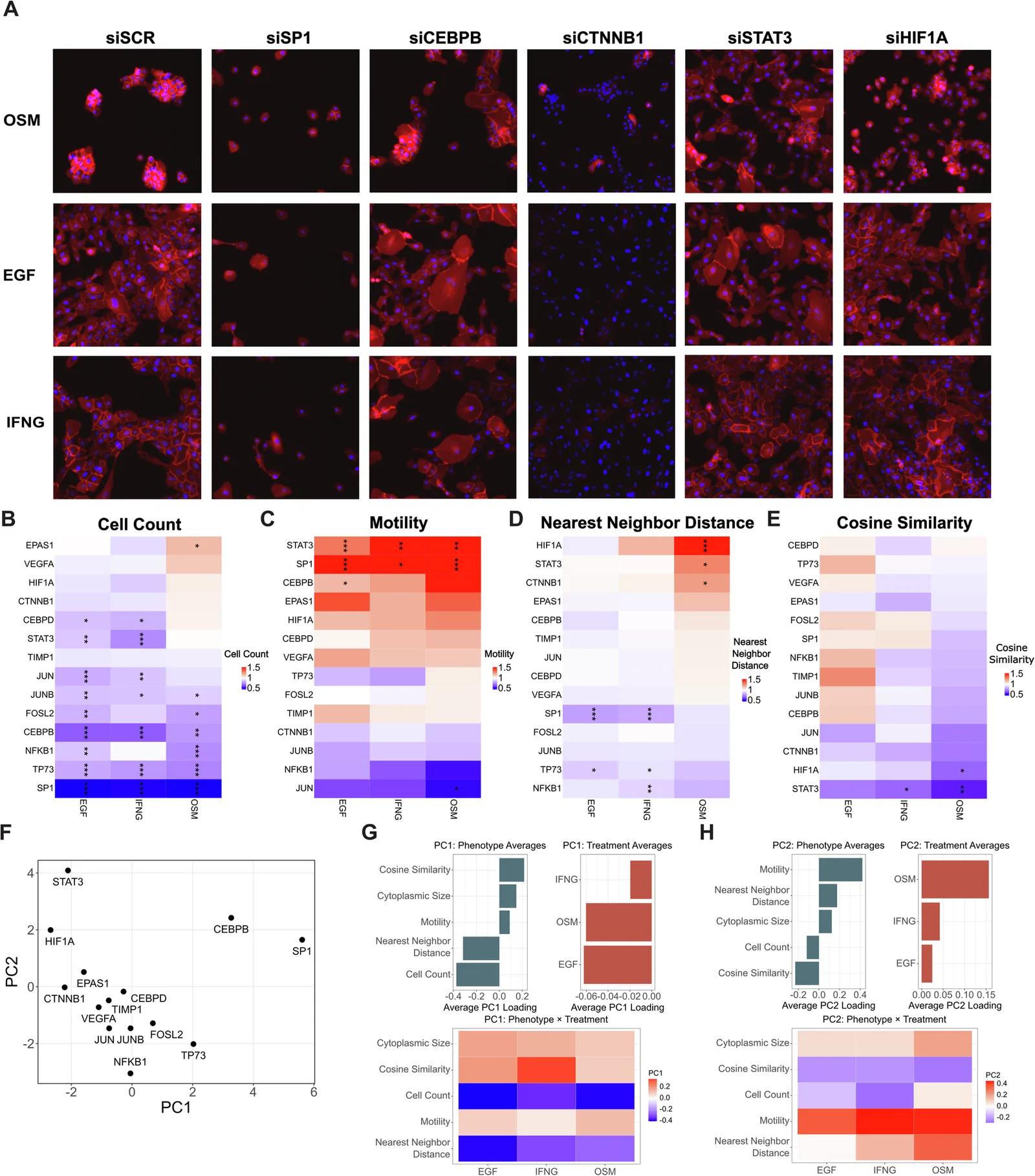
Quantification and visualization of siRNA experiment to identify drivers of OSM-induced CCM. **A** Representative immunofluorescent images from select knockdowns 48 hours after ligand treatment. **B**–**E** Quantification of cell phenotype induced by knockdown of nominated nodes (*n* = 3). Phenotypic scores have been normalized to the control (siSCR) condition for each ligand treatment. Statistical comparisons were performed using Student’s t-test. Significance is shown as follows: (*p < 0.05, **p < 0.01, ***p < 0.001). **F** Principal component analysis of phenotypic metrics across all ligand treatments. **G**–**H** PC1 and PC2 loadings: Bar plots show average PC loadings for each phenotype and each treatment. The accompanying tile plot displays PC loadings across all phenotype x treatment combinations, with color indicating loading magnitude and direction.

We hypothesized that nodes uniquely rewired by OSM play a causal role in mediating CCM. We tested this with siRNA knockdown of the 14 nominated nodes in MCF10A cells under three experimental conditions: OSM, EGF, and IFNG + EGF treatment. We reasoned that knockdowns producing phenotypic effects specific to the OSM condition, rather than broadly affecting MCF10A cells across conditions, would identify regulators functionally critical for OSM-induced CCM. Cells were analyzed by live-cell imaging and immunofluorescence assessment at 48 hours post-treatment (Fig. 3A), followed by quantitative assessment of cell count, MSD, nearest neighbor distance, and cosine similarity (Fig. 3B–E).

We identified knockdowns that selectively perturbed OSM-induced behaviors with principal component analysis (PCA) on the full set of phenotypic scores (Fig. 3F). PC1 was negatively weighted by cell count and nearest neighbor distance, indicating that high PC1 scores reflect reduced proliferation and disrupted cell clustering (Fig. 3G). Knockdowns of SP1 and CEBPB, which reduced cell number and altered cell shape across all ligand conditions, showed the strongest PC1 loadings. This PC captures general effects on cell viability or morphology, with no strong weighting from any specific ligand treatment, suggesting these regulators influence baseline epithelial functions rather than OSM-specific phenotypes (Fig. 3G). In contrast, PC2 was positively weighted by motility and negatively weighted by cosine similarity, suggesting that high PC2 scores reflect increased individual cell motility and reduced CCM (Fig. 3H). OSM treatment showed a strong positive weighting on PC2, indicating that knockdowns with high PC2 scores disproportionately disrupt OSM-induced phenotypes (Fig. 3H). siRNAs targeting STAT3 and HIF1A were enriched along this axis, implicating them as central OSM-specific regulators (Fig. 3F).

Consistent with their separation along PC2, knockdown of STAT3 and HIF1A increased nearest-neighbor distances and reduced cosine similarity under OSM treatment (Fig. 3D–E). siSTAT3’s disruption of clustering reinforces its role as a master regulator of OSM-induced behaviors, consistent with its centrality in the OSM network. HIF1A knockdown specifically reduced cosine similarity and increased nearest neighbor distance under OSM treatment, suggesting a critical role for HIF1A signaling in OSM-induced CCM. HIF1A has been linked to migratory and invasive phenotypes through its regulation of cytoskeletal and extracellular matrix remodeling genes^49,50^. The coordinated effects of HIF1A knockdown on clustering and cosine similarity support its function as a key regulator of OSM-induced CCM.

We observed several additional OSM-specific knockdown phenotypes. CTNNB1 knockdown increased nearest-neighbor distance only in the OSM condition, consistent with its role in maintaining cell-cell adhesion (Fig. 3D)^51^. Knockdown of JUN specifically reduced motility in OSM-treated cells, suggesting it may promote the motile component of CCM (Fig. 3C). EPAS1 (HIF2A) knockdown uniquely increased proliferation in the OSM context, an unexpected result given HIF2A’s reported role in promoting cell cycle progression, revealing a context-dependent relationship between this transcription factor and proliferation (Fig. 3B)^52^.

Supplementary scatterplots comparing phenotypic scores across ligand conditions further supported these observations, particularly demonstrating the differential effects of HIF1A and STAT3 knockdowns on OSM-induced phenotypes (Supplementary Fig. 3A–B). Comparing the magnitude of phenotypic changes under OSM versus IFNG revealed that most knockdowns produced larger effects under OSM (ratios >1). A binomial test confirmed this enrichment (*p* = 0.029), indicating that our comparative network approach successfully identified nodes that disproportionately impact phenotypes in the OSM context (Supplementary Fig. 3C). We annotated the OSM-specific network with the phenotypic effects observed in our siRNA experiment, labeling each node based on its functional impact (Supplementary Fig. 4A–B). This phenotype-annotated network provides a systems-level view of how distinct molecular regulators contribute to the diverse aspects of OSM-induced collective cell migration.

### HIF1A is a key mediator of OSM-induced CCM

**Fig. 4:**
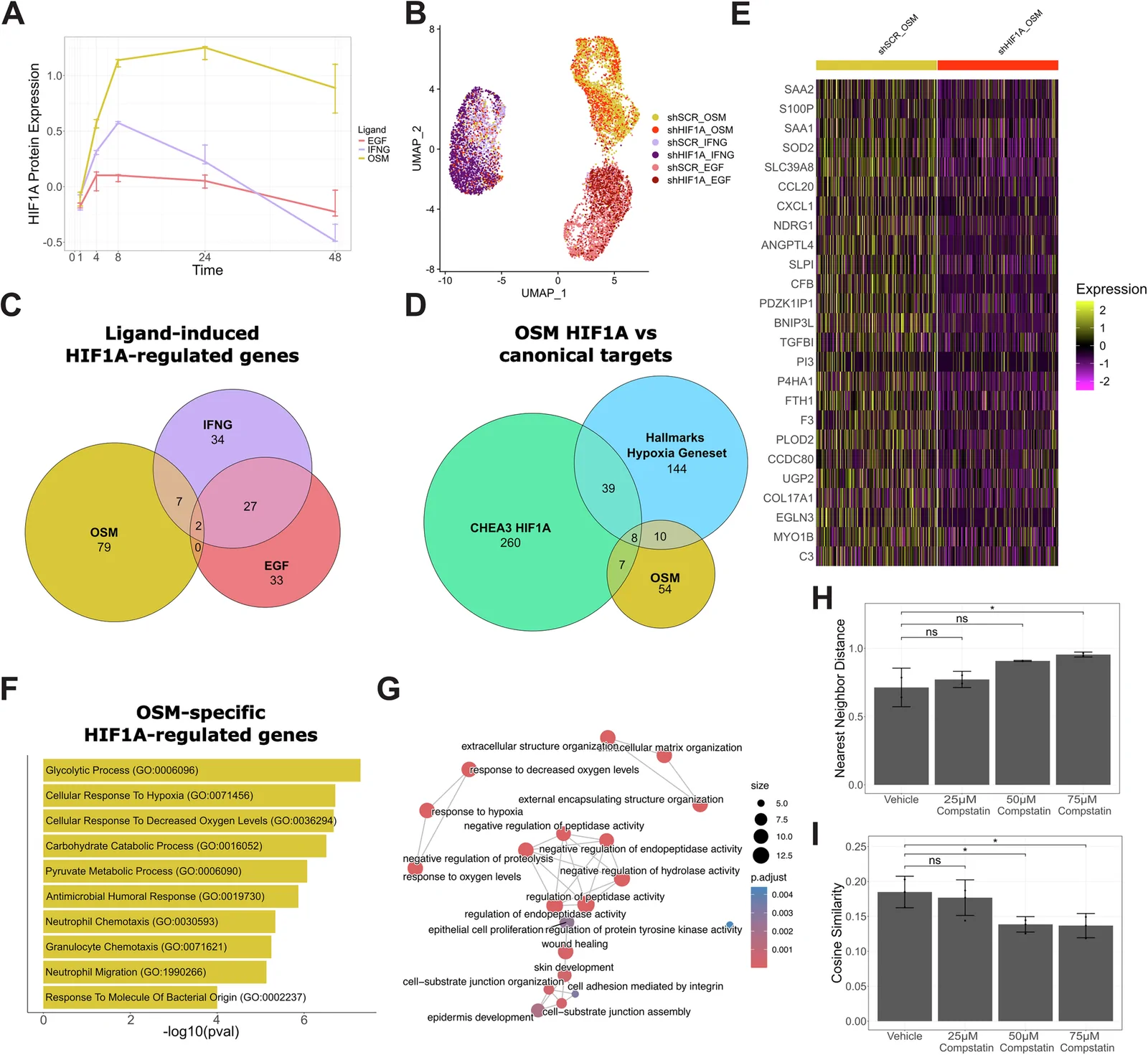
scRNAseq reveals HIF1A transcriptional program driving OSM-induced CCM. **A** OSM uniquely upregulates HIF1A protein expression over 48 hours of ligand treatment. Error bars represent standard deviation (*n* = 3). **B** UMAP projection of scRNAseq data indicates that transcriptional signals separate primarily by ligand treatment. **C** We identified HIF1A-regulated genes for each ligand condition by performing differential gene expression analysis between shSCR vs shHIF1A. Venn diagram represents a comparison of HIF1A-regulated gene sets under each treatment. **D** Comparison of unique OSM-HIF1A regulated genes to literature derived gene sets representing hypoxia and HIF1A transcription factor activity. **E** Relative expression of the top 20 differentially expressed unique OSM-HIF1A regulated genes. **F** We performed gene set enrichment analysis to identify Gene Ontology terms enriched in OSM-HIF1A regulated genes. Top ten most enriched gene sets are shown. Multiple comparison adjustments were made using the Benjamini-Hochberg method. **G** Enrichment map of Gene Ontology terms enriched in shSCR cells under OSM treatment. **H** Inhibition of complement signaling increases nearest neighbor distance in OSM treated cells and reduces cell clustering. Error bars represent the 95% confidence interval (*n* = 3). Statistical comparisons were conducted using Dunnett’s test (*p < 0.05). **I** Compstatin treatment reduces cosine similarity in OSM treated cells and inhibits CCM. Error bars represent the 95% confidence interval (*n* = 3). Statistical comparisons were conducted using Dunnett’s test (*p < 0.05).

Our functional analyses identified HIF1A as a key driver of OSM-induced CCM, leading us to next dissect its mechanistic contributions. Analysis of the RNA-seq and RPPA datasets described above showed sustained upregulation of HIF1A gene and protein expression over 48 hours of OSM treatment, an effect absent under EGF or IFNG (Fig. 4A, Supplementary Fig. 5A). Because STAT3 was also identified as a central regulator of OSM-induced CCM, we next investigated whether HIF1A activation occurs downstream of STAT3 signaling. Treatment of MCF10A cells with OSM and the STAT3 inhibitor Stattic reduced both phosphorylated STAT3 and HIF1A protein levels, indicating that STAT3 activity contributes to HIF1A induction in this system (Supplementary Fig. 5B, Supplementary Fig. 6A). These findings are consistent with prior studies demonstrating that STAT3 can directly regulate HIF1A transcription under normoxic conditions^46,53^.

To identify the transcriptional programs HIF1A activates in OSM-treated cells, we generated MCF10A cells with stable shRNA-mediated HIF1A knockdown (Supplementary Fig. 5C, Supplementary Fig. 6B). We treated shHIF1A and scramble control cells (shSCR) with OSM, EGF, or IFNG and performed single-cell RNA-seq at 24 hours. Assessment of HIF1A gene expression confirmed the knocked down in shHIF1A cells (Supplementary Fig. 5D). UMAP projection of scRNA-seq data showed that cells clustered primarily by ligand treatment, regardless of HIF1A knockdown status (Fig. 4B). To isolate transcriptional changes driven specifically by OSM-activated HIF1A, we compared differentially expressed genes in OSM-treated shHIF1A versus shSCR cells and excluded genes regulated by HIF1A under EGF or IFNG treatment. This analysis revealed 79 genes uniquely regulated by HIF1A in the OSM condition (Fig. 4C).

We more deeply examined the 79 OSM-specific HIF1A-regulated genes to prioritize candidate drivers of CCM. First, we asked whether these genes have already been linked to HIF1A and hypoxia signaling by comparison to Hallmark and ChEA3 gene sets; this confirmed overlap with 25 canonical hypoxia targets including NDRG1 and CA12 (Fig. 4D, Supplementary Fig. 5E–F)^54–57^. However, OSM-HIF1A also uniquely upregulated 54 additional genes not typically associated with hypoxia or canonical HIF1A transcriptional regulation, suggesting the activation of a specialized transcriptional program in the OSM context. These genes exhibited consistent upregulation in OSM-treated cells compared to other conditions (Fig. 4E). Second, gene set enrichment analysis (GSEA) showed that these 79 genes were enriched for glycolysis and metabolic reprogramming pathways, including “glycolytic process”, “carbohydrate catabolic process”, and “pyruvate metabolic process” (Fig. 4F)^58^. We also observed enrichment of hypoxia-related gene sets, including “cellular response to hypoxia” and “cellular response to decreased oxygen levels.” These findings align with HIF1A’s well-documented role in promoting glycolysis and mediating the cellular response to hypoxia, confirming that HIF1A retains aspects of its canonical functionality in the OSM condition^59^.

In addition to metabolic and hypoxia-related processes, we observed enrichment of immune-related pathways, particularly those involving neutrophil migration and chemotaxis. Because neutrophils engage in collective migration, we hypothesized that this transcriptional signature may relate to the observed OSM-induced CCM phenotype^60–62^. Closer examination of the enrichment map linking these gene sets revealed HIF1A-regulated genes involved in complement signaling and regulation of peptidase activity (Fig. 4G), a pathway associated with cell adhesion and collective migration in other biological contexts^63–65^. Consistent with this, we observed that the central complement component C3 is upregulated in OSM-treated control cells relative to HIF1A knockdown, and that genes associated with the alternative complement pathway are similarly enriched, supporting activation of complement-related programs downstream of HIF1A (Fig. 4G, Supplementary Fig. 5G).

To test whether complement signaling functionally contributes to OSM-induced CCM, we treated cells with OSM and Compstatin, an inhibitor of C3, the central hub of complement activation that drives downstream effector pathways^66^. Live-cell imaging showed that Compstatin treatment reduced clustering (Fig. 4H**(**25 µM *p* = .630, 50 µM *p* = .056, 75 µM *p* = .028) and cosine similarity (25 µM *p* = .646, 50 µM *p* = .027, 75 µM *p* = .023) in a dose-dependent manner, mirroring phenotypes observed with HIF1A knockdown (Fig. 4I, Supplementary Movie 1,2). These findings establish a mechanistic link between OSM signaling, HIF1A activation, complement signaling, and CCM. The HIF1A-dependent transcriptional program induced by OSM encompasses canonical hypoxia targets as well as genes involved in immune and complement-related pathways, consistent with a broader regulatory role for HIF1A in epithelial collective migration.

### OSMR and its downstream transcriptional programs are active in human breast tumors and associated with poor prognosis

**Fig. 5:**
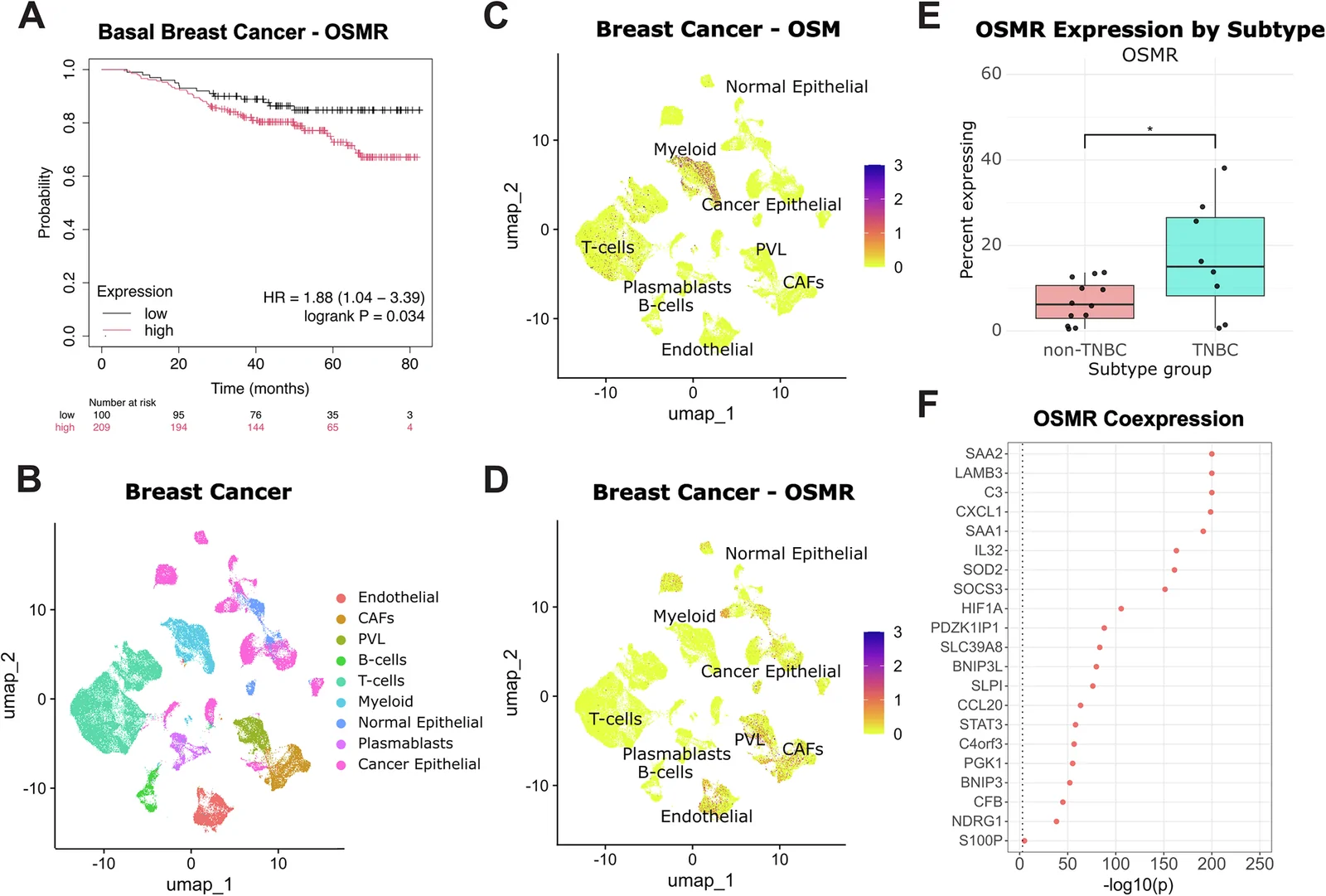
The OSM–HIF1A signaling axis is active in basal-like breast cancer and associated with poor prognosis. **A** Kaplan–Meier survival analysis of patients with basal-like breast cancer stratified by OSMR expression (TCGA-BRCA, *n* = 309). High OSMR expression is associated with reduced overall survival (log-rank *P* = 0.034). **B** UMAP of single-cell RNA-seq data from human breast tumors annotated by cell type. **C** UMAP showing OSM is primarily expressed by myeloid cells in breast cancer tumors. **D** UMAP showing OSMR expression in stromal and epithelial tumor compartments, including epithelial cancer cells. **E** Boxplot showing the percentage of epithelial cells expressing OSMR in individual patients, comparing triple-negative breast cancer (TNBC) to non-TNBC tumors. TNBC tumors have a higher proportion of OSMR-positive epithelial cells (*p < 0.05). **F** COTAN analysis of OSMR coexpression within cancer epithelial cells shows robust coexpression of mechanistic signature. Dotted line indicates significance threshold (*p* < 0.01).

Given the role of CCM in cancer invasion and metastasis, we hypothesized that OSM signaling and its downstream effectors contribute to poor outcomes in breast cancer patients. High OSMR expression was significantly associated with reduced overall survival in patients with PAM50-classified basal breast cancers (log-rank *P* = 0.034) (Fig. 5A)^67,68^. To identify the cell types driving or responding to OSM signaling, we analyzed publicly available single-cell RNA-sequencing data from clinical breast cancer samples^69^. OSM expression was primarily restricted to tumor-infiltrating myeloid cells (Fig. 5C), whereas OSMR expression was enriched in multiple non-myeloid cell types, including a subset of cancer epithelial cells, endothelial cells, fibroblasts, and perivascular cells (Fig. 5D). These findings suggest that OSM signaling is active in the tumor microenvironment and may influence both malignant and stromal components, potentially driving aggressive tumor behavior.

To further explore the role of OSMR signaling in malignant epithelial cells, we stratified tumors by molecular subtype. Epithelial cells from triple-negative breast cancer (TNBC) tumors were more likely to express OSMR (*p* = 0.029) and C3 (*p* = 0.001) compared to other subtypes (Fig. 5E, Supplementary Fig. 7A). Within each subtype, the percentage of epithelial cells expressing OSMR varied significantly by patient, reflecting inter-patient heterogeneity (Supplementary Fig. 7B). Finally, we assessed whether the components of the OSM–STAT3-HIF1A transcriptional program are coexpressed within individual cancer epithelial cells using COTAN analysis^70^. We examined coexpression between OSMR, STAT3, and the top 20 HIF1A-regulated genes (including HIF1A). This analysis revealed that in malignant epithelial cells, all genes in this set are significantly coexpressed with OSMR, indicating coordinated activation of this transcriptional program in malignant epithelial cells (Fig. 5F).

Together, these results demonstrate that the OSM–HIF1A signaling axis and its downstream transcriptional programs are active in basal-like breast tumors. The enrichment of an OMSR gene program in malignant epithelial cells and the association of this gene program with poor survival suggests that this cytokine-driven collective migration mechanism may contribute to disease progression in breast cancer. These observations extend our findings to human disease and suggest that targeting the OSM-HIF1A axis could have therapeutic value for the subset of TNBC patients with high OSMR expression.

## Discussion

Cell migration is a key process in various physiological and pathological contexts, including tissue development, wound healing, and cancer metastasis. CCM, in which groups of cells move together as a cohesive unit, plays a critical role in maintaining tissue architecture and facilitating invasion during cancer metastasis^1^. The mechanisms driving CCM are complex, involving interactions between signaling pathways, cytoskeletal dynamics, and cell-cell communication. In this study, we explored how the cytokine OSM drives CCM in mammary epithelial cells. Through network analysis and experimental validation, we identified HIF1A and STAT3 as central regulators of OSM-induced CCM.

Our results demonstrate that OSM induces a unique transcriptional program that drives CCM, with HIF1A and STAT3 acting as key regulators. Using shRNA, we mapped the functional role of these nodes in modulating phenotypic responses. HIF1A knockdown significantly impaired OSM-induced CCM, confirming its central role in this process. Similarly, STAT3, a canonical mediator of cytokine signaling, was required for the induction of OSM-dependent clustering and migration^71^. Together, these findings suggest that OSM promotes a coordinated cellular response that integrates hypoxic and inflammatory cues to drive collective migration.

HIF1A is classically known for its role in regulating cellular responses to hypoxia, including metabolic reprogramming, survival, and motility^67^. More recently, HIF1A has been implicated in promoting partial epithelial-to-mesenchymal transition (EMT), a transcriptional and phenotypic program that enhances cellular plasticity, motility, and invasiveness while preserving epithelial characteristics such as cell-cell junctions^20,68^. Our data are consistent with this model; OSM treatment upregulated HIF1A expression and activated downstream gene programs that resulted in cells retaining intercellular junctions while acquiring migratory capacity^72,73^. These findings may be particularly important in cancer biology, where collective invasion has been increasingly recognized as a driver of metastasis^74–76^. Prior studies have reported regulatory interactions between HIF1A and SNAI1, including evidence that HIF1A can directly regulate SNAI1 expression under hypoxic conditions and that these factors may cooperatively regulate transcriptional programs associated with EMT and migration^53,77,78^. Consistent with this, we observed upregulation of SNAI1 following OSM treatment; however, we did not directly assess the regulatory relationship between HIF1A and SNAI1 in this system.

Notably, HIF1A is classically regulated through oxygen-dependent protein stabilization^79^; however, our experiments were performed under normoxic conditions, suggesting an alternative mechanism of activation. Prior studies have shown that cytokine signaling pathways, including IL-6/STAT3 and OSM, can directly induce HIF1A transcription independent of hypoxia^46,53^. STAT3 was reported to directly bind the HIF1A promoter and promote its expression^53^. These findings are consistent with our observations that HIF1A is transcriptionally upregulated following OSM treatment and that this upregulation is dependent on STAT3 activation. More broadly, our findings suggest that IL-6 family cytokines may share a common mechanism of HIF1A activation, whereby cytokine signaling induces STAT3, leading to transcriptional upregulation of HIF1A and engagement of hypoxia-associated gene programs under normoxic conditions.

An additional finding in our study was the enrichment of complement-related immune pathways in HIF1A-regulated genes. This prompted us to investigate whether complement activation functionally contributes to OSM-induced migration. Using Compstatin, a C3-specific inhibitor, we observed a marked reduction in both clustering and motility, mirroring the effects of HIF1A knockdown. Consistent with this, we found that C3 expression is upregulated in a HIF1A-dependent manner following OSM treatment; prior studies have shown that MCF10A cells are capable of secreting C3, supporting the feasibility of local complement activity in this system^80^. Together, these finding suggest that complement signaling acts downstream of HIF1A and contribute to cellular behaviors necessary for CCM. The identification of complement as a candidate pathway provides insight into how immune and hypoxic signals may converge to influence collective migration and suggests that targeting complement signaling could modulate these behaviors in disease contexts. However, the precise mechanism by which complement is activated remains unclear. Possible modes of action include modulation of coattractive autocrine/paracrine signaling through complement receptors^64^, activation of chemotaxis signals and their downstream effectors^63^, or indirect effects on integrin regulation and cell–cell adhesion^65^. Further work will be required to define how complement is activated in this context and to delineate its functional contribution relative to other HIF1A-dependent pathways. Direct measurement of C3 secretion and downstream complement activation in OSM-treated MCF10A cells will be important to confirm functional engagement of this pathway beyond the transcriptional evidence presented here.

In addition, gene set enrichment analysis revealed a strong upregulation of glycolysis-related genes, further linking HIF1A to metabolic reprogramming in OSM-treated cells. While glycolytic shifts are a well-known feature of the hypoxic response, our data raise the possibility that OSM-induced HIF1A activity may contribute to broader metabolic changes in tumors^59,81^. These shifts could reflect a reallocation of resources to meet the energetic and biosynthetic demands of collective cell migration^82,83^. More broadly, these findings suggests that OSM, via HIF1A, may coordinate both migratory and metabolic responses.

Motivated by our in vitro findings, we investigated the relevance of this pathway in breast cancer by analyzing publicly available scRNA-seq data^69^. We found that OSM is primarily expressed by tumor-infiltrating myeloid cells, whereas OSMR, the cognate receptor, is expressed by a subset of epithelial tumor cells. Notably, OSMR expression was highest in triple-negative breast cancer (TNBC) tumors, a subtype molecularly aligned with basal breast cancer and associated with poor prognosis^84^. This suggests that OSM-driven signaling may be active in aggressive cancer subtypes. Supporting this, we found that OSMR expression is negatively associated with survival in patients with basal breast cancer. Our analysis also revealed significant coexpression between OSMR and components of the HIF1A-associated gene program, including HIF1A itself, in malignant breast cancer epithelial cells. These findings are consistent with the presence of an active OSM-HIF1A-complement axis in vivo. However, we did not observe a significant association between the HIF1A-regulated transcriptional signature and survival in bulk tumor datasets. Because many components of this signature are associated with immune and complement processes, bulk transcriptomic analyses may obscure epithelial cell–specific pathway activity by capturing expression from diverse stromal and immune populations. Future studies using spatial transcriptomics or epithelial cell–restricted analyses may better define the prognostic significance of this program within malignant cells. While we have not directly assessed the role of the OSM axis in primary versus metastatic disease, the link between CCM and metastatic dissemination^85^ raises the possibility that OSM-induced CCM could contribute to metastatic progression. In this context, the association between high OSMR expression and poor survival further supports the potential clinical significance of this pathway.

While our study provides a comprehensive characterization of OSM-induced CCM in MCF10A cells, it has several limitations. Most notably, our findings are based on a non-transformed mammary epithelial cell line. Although this model offers a controlled environment to study cytokine responses, future efforts could extend these observations to cancer-derived organoids^86^, xenografts^87^, or patient samples^88^ to evaluate how OSM-driven CCM operates in diverse tumor microenvironments. In addition, while we focused on transcriptional changes, CCM is also regulated by post-translational modifications^89,90^, cytoskeletal rearrangements^91^, and dynamic cell-cell interactions^92^, which could be further explored with advanced imaging approaches. Additionally, our analysis focused primarily on the transcriptional consequences of HIF1A activation, yet HIF1A also modulates various proteomic signaling pathways that could influence CCM^1^. While we observed increased HIF1A protein levels following OSM treatment, our data do not distinguish whether this reflects protein stabilization or strictly transcriptional upregulation, and both mechanisms may contribute to HIF1A activation in this context. Pathways like mTOR signaling, integrin-mediated adhesion, and autophagy, which are also regulated by HIF1A, may contribute to CCM by altering cellular motility, adhesion, and metabolic support for migration^49,93,94^. Further investigation into how HIF1A coordinates these signaling pathways will provide a more comprehensive understanding of its role in CCM.

In conclusion, our findings identify HIF1A as a key mediator linking inflammatory cytokine signaling and CCM, processes relevant to cancer progression^20,76^. Consistent with this, we observed that OSMR expression negatively correlates with survival in basal breast cancer, and that breast epithelial cells frequently coexpress OSMR, HIF1A, and our hypoxia-associated gene signature. Our results are consistent with prior work implicating OSM in cancer progression and provide a foundation for future studies to investigate how these pathways contribute to cancer metastasis and tissue remodeling.

## Methods

### MCF10A cell culture

Cell culture and ligand perturbation experiments were carried out according to the methods described by Gross et al.^23^. Briefly, for routine cell growth and passaging, MCF10A cells were maintained in a growth medium consisting of DMEM/F12 (Invitrogen #11330-032), 5% horse serum (Sigma #H1138), 20 ng/ml EGF (R&D Systems #236-EG), 0.5 µg/ml hydrocortisone (Sigma #H-4001), 100 ng/ml cholera toxin (Sigma #C8052), 10 µg/ml insulin (Sigma #I9278), and 1% Pen/Strep (Invitrogen #15070-063). For perturbation studies, a growth factor-free medium was used, which included DMEM/F12, 5% horse serum, 0.5 µg/ml hydrocortisone, 100 ng/ml cholera toxin, and 1% Pen/Strep.

MCF10A cells were cultured until they reached 50-80% confluence in growth medium, followed by detachment using 0.05% trypsin-EDTA (Thermo Fisher Scientific #25300-054). After detachment, 6000 cells were seeded into collagen-1 (Cultrex #3442-050-01) coated 24-well plates (Thermo Fisher Scientific #267062) with growth medium. Six hours later, the cells were washed with PBS, and growth factor-free medium was added. The cells were then incubated for 18 hours in the new medium. Afterward, cells were treated with either 10 ng/ml EGF (R&D Systems #236-EG), 10 ng/ml OSM (R&D Systems #8475-OM), or 20 ng/ml IFNG (R&D Systems #258-IF) + 10 ng/mL EGF. IFNG was administered together with EGF because, unlike OSM, IFNG alone does not support MCF10A cell proliferation^23,34^.

### Live-cell imaging

Live-cell imaging was conducted using the Incucyte S3 microscope (Essen BioScience, #4647), with images captured every 30 minutes for a duration of up to 48 hours. All experiments were performed using three biological replicates. The resulting live-cell image stacks were processed by first registering them with a custom Fiji script and then segmented using CellPose v3.01^95,96^. Image tracking was performed using the Baxter Algorithms pipeline^97^.

Subsequent analysis of the cell tracking data was conducted within RStudio^98^. Cell counts were calculated by determining the number of cells per field, normalized by the initial T0 count for each field. Nearest neighbor distances were computed by measuring the Euclidean distances in pixels from the centroid of each cell to the centroid of the second nearest cell within the imaging field. To adjust for variations in cell count, the average nearest neighbor distances for each image were normalized by the expected mean distance to the nearest neighboring cell under a random cell distribution model^99^. Cell motility was assessed by excluding tracks with jumps greater than 200 pixels in a 30-minute interval, as these tracks likely represent tracking errors. Motility was quantified as the slope of the mean squared displacement (MSD) over time intervals ranging from 30 minutes to 6 hours^100^. This slope was determined by constructing a linear model comparing MSD to time intervals, and is proportional to the diffusion coefficient associated with Brownian motion^100^. Finally, collective cell migration was estimated by calculating the cosine similarity between displacement vectors of the 10 nearest cells every 30 minutes. Statistical comparisons of phenotypic scores between ligand conditions was performed using Dunnett’s test, with EGF serving as the control^101^.

### Immunoflourescent Imaging

For all immunofluorescent experiments, cells were fixed in 4% formaldehyde after 48 hours of ligand treatment. Cells were blocked for 1 hour in a PBS solution (5% Normal Goat Serum (Cell Signaling #5425), 0.3% Triton X (Thermo Fisher Scientific #85111), then incubated overnight with the primary conjugated antibody (Cell Signaling #2677) at a 1:500 dilution. Cells were washed thoroughly then counterstained with 0.5 mg / mL DAPI (PromoKine PD-CA707-40043). Imaging was performed using the InCell 6000 (GE Healthcare).

### RNAseq, RPPA Data, and Cyclic IF

The bulk RNA sequencing (RNAseq), reverse phase protein array (RPPA) data, and cyclic immunofluorescence (cycIF) data after ligand treatments were generated in a prior investigation^23^. For detailed information on the methodologies used for data generation, normalization, and integration, please refer to the original publication.

### Causal pathway analysis and network analysis

Causal Pathway was utilized to construct gene and protein interaction networks^37^. The input to the algorithm was the integrated features from RNAseq, RPPA, and cyclic IF datasets. Data from proteomic assays were favored for features shared between assays. Only features with a log-fold change (LFC) of 1 or greater compared to the time zero control were included in the analysis. The generated networks were subsequently integrated and analyzed in Cytoscape^38^.

To identify gene/protein nodes for experimental testing, a comparative analysis was performed between the IFNG and OSM networks. Each node was assigned a score based on network rewiring, when comparing the OSM network to the IFNG network using Dynet^39^. This score reflects the number of interaction changes (edge gains or losses) required to transform a node’s connectivity between conditions. This score was then scaled according to the LFC in the OSM condition. The fourteen nodes with the highest comparative network importance scores were selected for experimental validation.

### siRNA screen and drug experiments

Reverse transfection of MCF10A cells was performed according to DharmaFECT Transfection vendor protocols. Cells were seeded in collagen coated 24-well plates with growth media as previously described (see methods: MCF10A cell culture). After 6 hours in culture, media was replaced with 25 nM siRNA and 0.5 mL Lipofectamine™ RNAiMAX Transfection Reagent (Thermo Fisher Scientific #13778075) per well in antibiotic free media. After 48 hours of transfection, cells were washed thoroughly with PBS and replaced with assay media and indicated ligand treatments. The siRNA screen was run in triplicate. A full list of siRNAs used in the study is included (Supplementary Table 1). Immediately after ligand treatment, live-cell imaging and phenotypic quantification was performed as described previously (see Methods: Live-Cell Imaging). Statistical comparisons between phenotypic scores were performed by pairwise Student’s *t*-tests between knockdown conditions and the siSCR condition for each ligand treatment^102^. Principal Component Analysis (PCA) was performed on phenotype quantifications using the prcomp function from the stat R package (3.6.2)^98^.

For drug experiments, cells were cultured and plated as previously described (see Methods: MCF10A cell culture). Concurrent to ligand treatment, cells were incubated with either vehicle, or 25 µM - 75 µM Compstatin (Selleckchem #S8522) or 5 µM Stattic (MedChemExpress #HY-13818). Statistical comparisons between treated and untreated conditions were conducted using Dunnett’s test^101^. Significance was defined as *p* < 0.05.

### shRNA integration

Plasmid constructs for non-targeting SMARTvector Lentiviral shRNA and shRNA sequences targeting HIF1A were purchased from Horizon Discovery (Horizon Discovery # VSC11287, #3SH11243-02EG3091). Expression of the shRNA was driven by the EF1A promoter and contained Puromycin resistance and TurboGFP elements to ensure proper integration. *E. Coli* containing the plasmid constructs were inoculated into LB broth medium containing 100 μg/mL carbenicillin and incubated at 37 C for 18 hours with shaking. Plasmids were extracted using the HiSpeed Plasmid Midi Kit (Qiagen #12643).

Plasmids were then packaged into lentiviral particles by transfecting 293 T human embryonic kidney cells (ATCC #CRL-3216) with the shRNA plasmid construct and lentiviral package plasmids (Addgene #8454, #12263) using LF2000 transfection reagent (Invitrogen #11668019). Virus containing media was collected through a 0.45 mm low-protein binding filter (Sterlitech #PES4547100). MCF10A cells were then transduced with virus containing media and 10 mg/mL polybrene. After a four-hour transduction, media was replaced with growth media, and shRNA containing cells were selected with 0.5 mg/mL puromycin. Expression of shRNA was confirmed using fluorescent imaging of GFP on the Incucyte S3 microscope (Essen BioScience #4647).

### Immunoblotting

HIF1A, STAT3, and phospho-STAT3 were detected in WT cells, and cells transduced with shRNA through immunoblotting. After 24 hours of treatment with OSM or EGF, cells were lysed in hot Laemmli buffer (Santa Cruz Biotechnology #sc-286962). Cell lysates were boiled at 95 C for 10 minutes, then total protein concentration was determined and normalized through BCA assay (Thermo Fisher Scientific #23225). 25 µL of cell lysate was loaded onto gels (Invitrogen #NP0321BOX) and proteins were separated at 200 V for 45 minutes then transferred at 15 V overnight at 4 C. After transfer the membrane was blotted with TBST and 5% dry milk for 1 hour, then probed for HIF1A (1:1000, BD Biosciences #610959), STAT3 (1:1000, Cell Signaling #9139), and phospho-STAT3 (Tyr705) (1:2000, Cell Signaling #9145) and secondary antibody at a concentration of 1:10000 (Jackson Immunoresearch #715-035-150, #111-035-144). Chemiluminescence was imaged on the Alpha Innotech FluorChem Imaging System (#22424).

### scRNA-seq library preparation and sequencing

All cell lines and ligand conditions were multiplexed using Hashtag Oligonucleotide barcoding (TotalSeq-A, Biolegend #399907) according to the vendor’s recommendations. The mRNA library was created using the Chromium Single Cell 3ʹ GEM, Library & Gel Bead Kit v3 (10X Genomics #1000092) and then sequenced using an Illumina NovaSeq with a depth of 800 million reads. Short read sequencing assays were performed by the OHSU Massively Parallel Sequencing Shared Resource.

### scRNA-seq data processing and analysis

Raw files were converted to FASTQ format with bcl2fastq (version 2.20.0.445). Cellranger count was used to align reads to the GRCh38 transcriptome (GCF_000001405.40). The R package deMULTIplex was used to demultiplex the hash-tagged samples and assign cell lines and treatments to cells^103^. Seurat (4.0.5) was used to perform variable feature identification, dimensionality reduction, unsupervised clustering, visualization, and differential gene expression^104^. Differential expression analysis was performed using the FindMarkers function of Seurat with default parameters. GSEA was performed with the R package clusterProfiler on Gene Ontology Biological Process gene sets using significantly upregulated genes compared to the shSCR control (LFC > 0.5, *p* < 0.05)^105^.

### Survival analysis

To assess the prognostic significance of OSMR and OSM-HIF1A target gene expression, we utilized the Kaplan–Meier Plotter (KmPlot) web tool, which integrates public RNA sequencing data from breast cancer patient cohorts^106,107^. Patients were stratified into two groups based on gene expression levels using the tool’s automatic cutoff selection algorithm, and overall survival was compared using the log-rank test.

### Breast cancer single-cell RNA sequencing analysis

To investigate OSM pathway activity within the tumor microenvironment, we analyzed a publicly available single-cell RNA sequencing atlas of 26 primary human breast tumors^69^. Processed expression matrices were analyzed using Seurat (4.0.5)^104^. The pipeline included variable feature identification, dimensionality reduction, unsupervised clustering, visualization, and differential gene expression analysis. Differential expression was performed using Seurat’s FindMarkers function, applying a p threshold of <0.01 and a log2 fold-change cutoff > 1. Cell type and breast cancer subtype annotations were obtained from the original publication. To quantify transcriptional coordination of OSM-HIF1A genes in malignant epithelial cells, we calculated coexpression scores and estimated ps using the COTAN package^70^. Genes with a coexpression *p* < 0.01 were considered significantly coexpressed.

## Supplementary information

Supplementary information

Supplementary Movie 1

Supplementary Movie 2

## Data Availability

Raw live-cell imaging and IF imaging data of MCF10A cells treated with siRNA knockdown and OSM, EGF, or IFNG are available Zenodo: 10.5281/zenodo.17101147, 10.5281/zenodo.17122918, 10.5281/zenodo.17136555, 10.5281/zenodo.17136574. Single-cell RNA-seq data generated in this study have been deposited in the Gene Expression Omnibus (GEO) under accession number: GSE307221.

## Code availability

All analysis scripts, along with a complete list of R packages and versions used, are available at: https://github.com/mcleania/MCF10A_OSM_HIF1A. Any additional information required to reanalyze the data reported in this paper is available from the lead contact upon request.
