# Supplementary material for: Oncostatin M orchestrates collective epithelial migration via HIF1A activation
Source: NPJ Syst Biol Appl. 2026 Jul 6;12:162. doi: 10.1038/s41540-026-00778-0 (PMC13720081; doi:10.1038/s41540-026-00778-0)
Supplement: Supplementary file 1 [file 41540_2026_778_MOESM1_ESM.pdf]

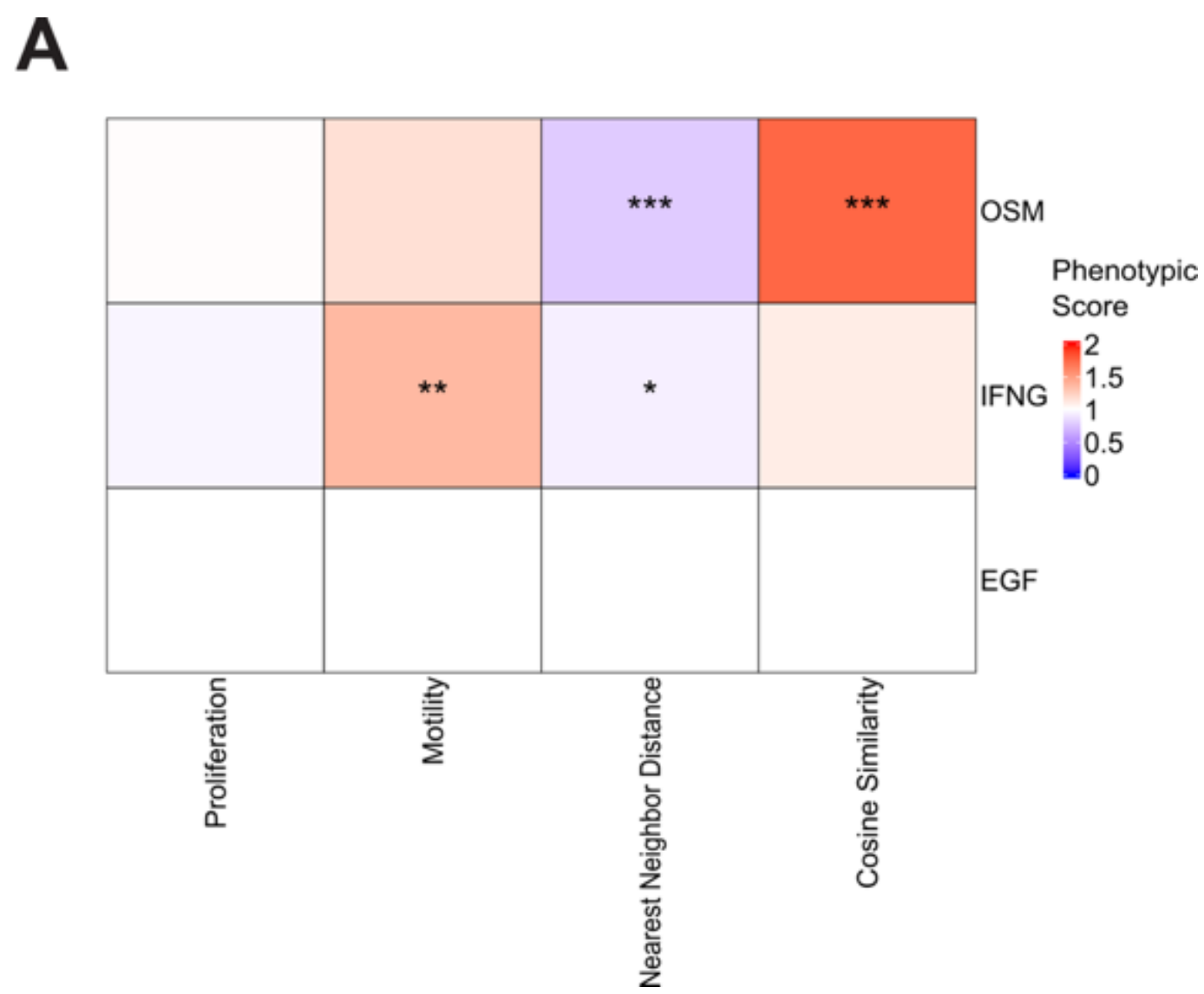

Supplementary Figure 1: Statistical comparison of phenotypic metrics.

(A) Statistical comparison of the quantifications of cellular proliferation, motility, nearest neighbor distance, and cosine similarity. Phenotypic scores have been normalized to the EGF condition. Dunnett's test was employed to compare phenotypic scores to the EGF control (n=3). Significance is shown as follows: (* p-value < .05, ** p-value < .01, *** p-value < .001).

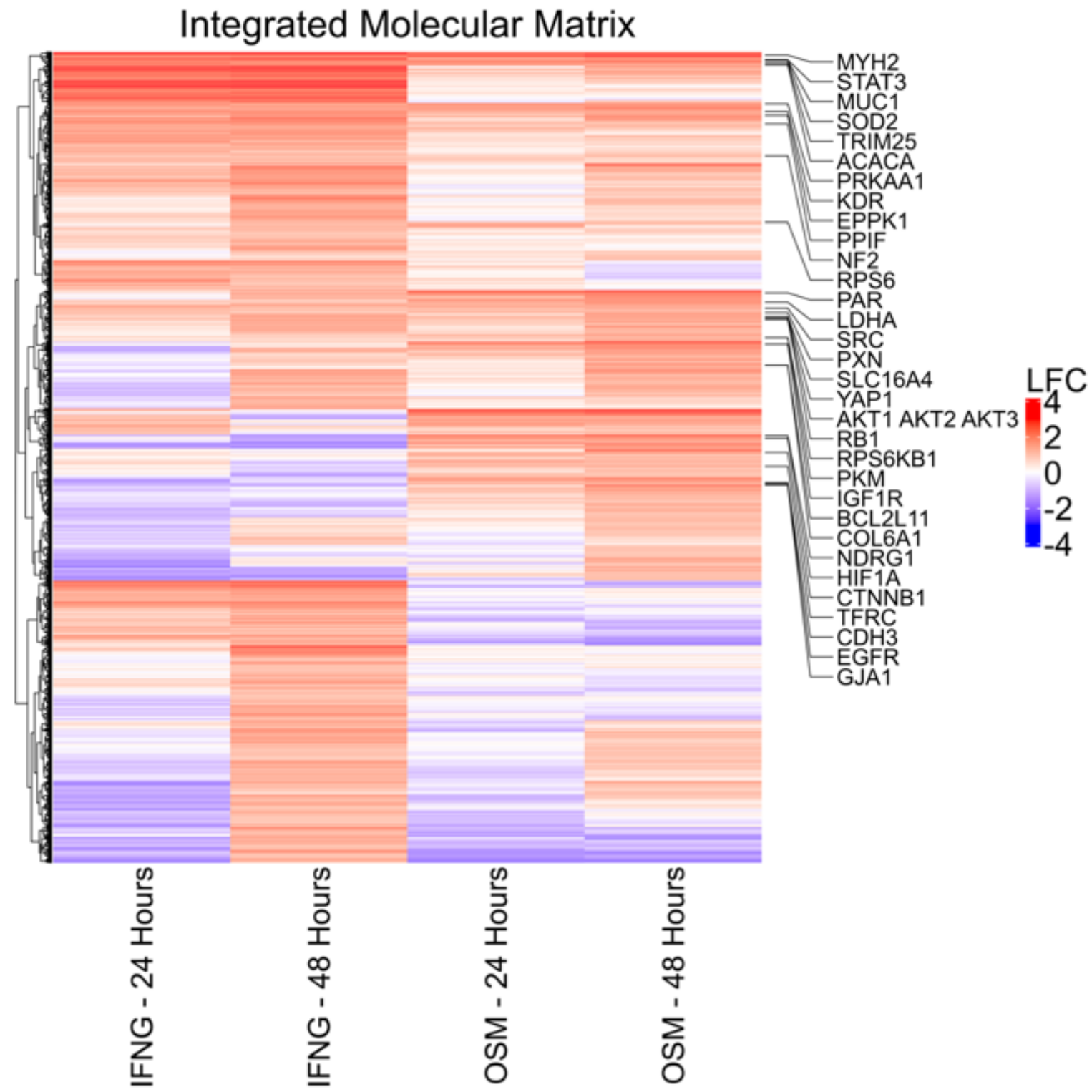

Supplementary Figure 2: Integrated and filtered molecular data collected from OSM and IFNG treated cells

Proteomic (RPPA, CycIF) and transcriptional (RNAseq) data was collected from MCF10A cells treated with OSM or IFNG for 24 and 48 hours (n=3). The data from each assay was normalized to the Time 0 control, filtered for differentially expressed features (LFC > 1, p-value < .05 adjusted by FDR) and then integrated into a combined dataset. Proteomic features from the RPPA assay are labelled.

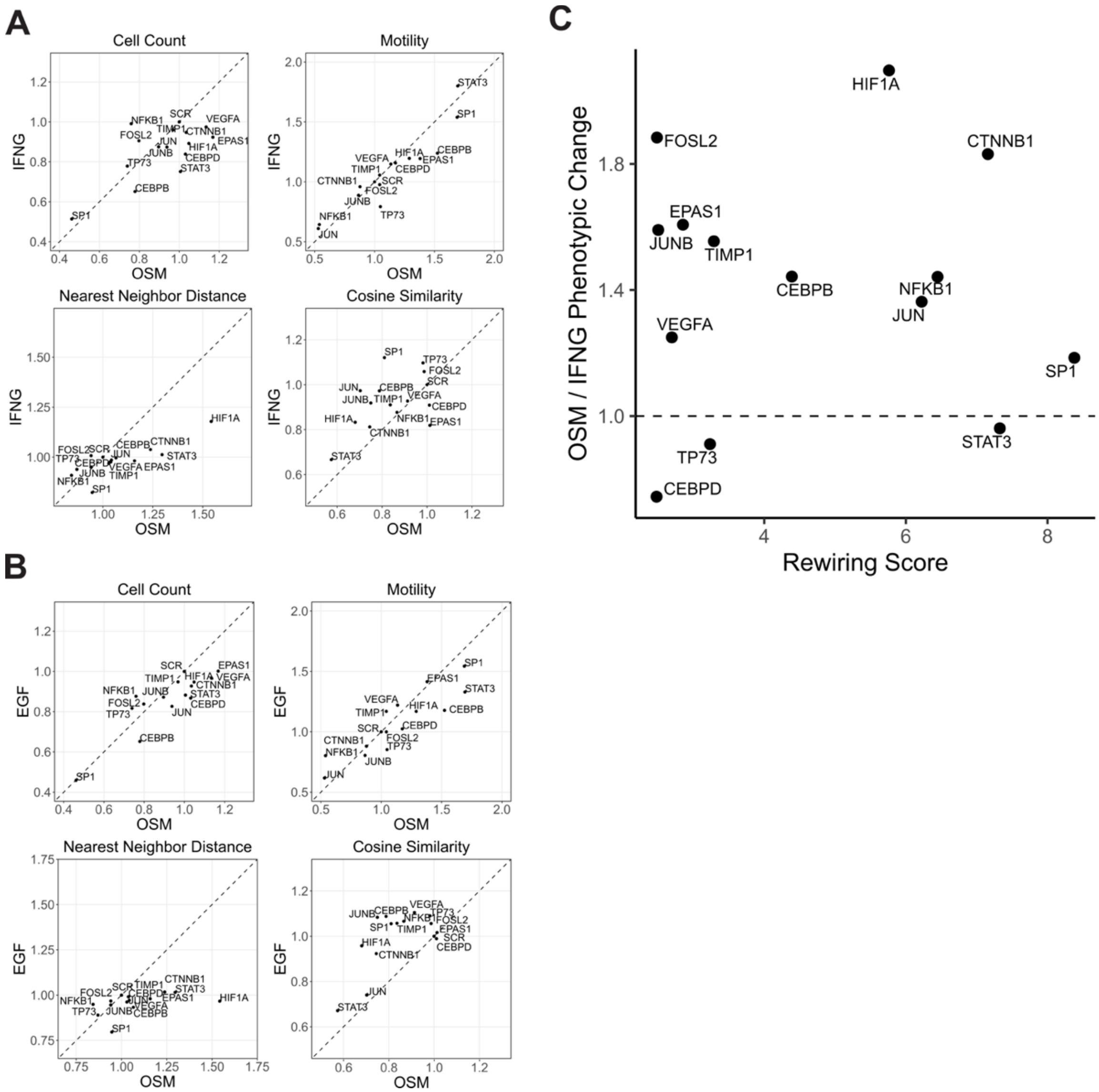

Supplementary Figure 3: Comparison of phenotypic effects of knockdown between ligand conditions

(A-B) Comparison of the phenotypic effects of knockdown on OSM and (A) IFNG or (B) EGF treated cells. (C) Scatterplot comparing the rewiring score to the overall magnitude of phenotypic change for each knockdown relative to the scramble control. Nominated nodes were more likely to exert greater effects under OSM treatment compared to IFNG (binomial test, $p = 0.029$).

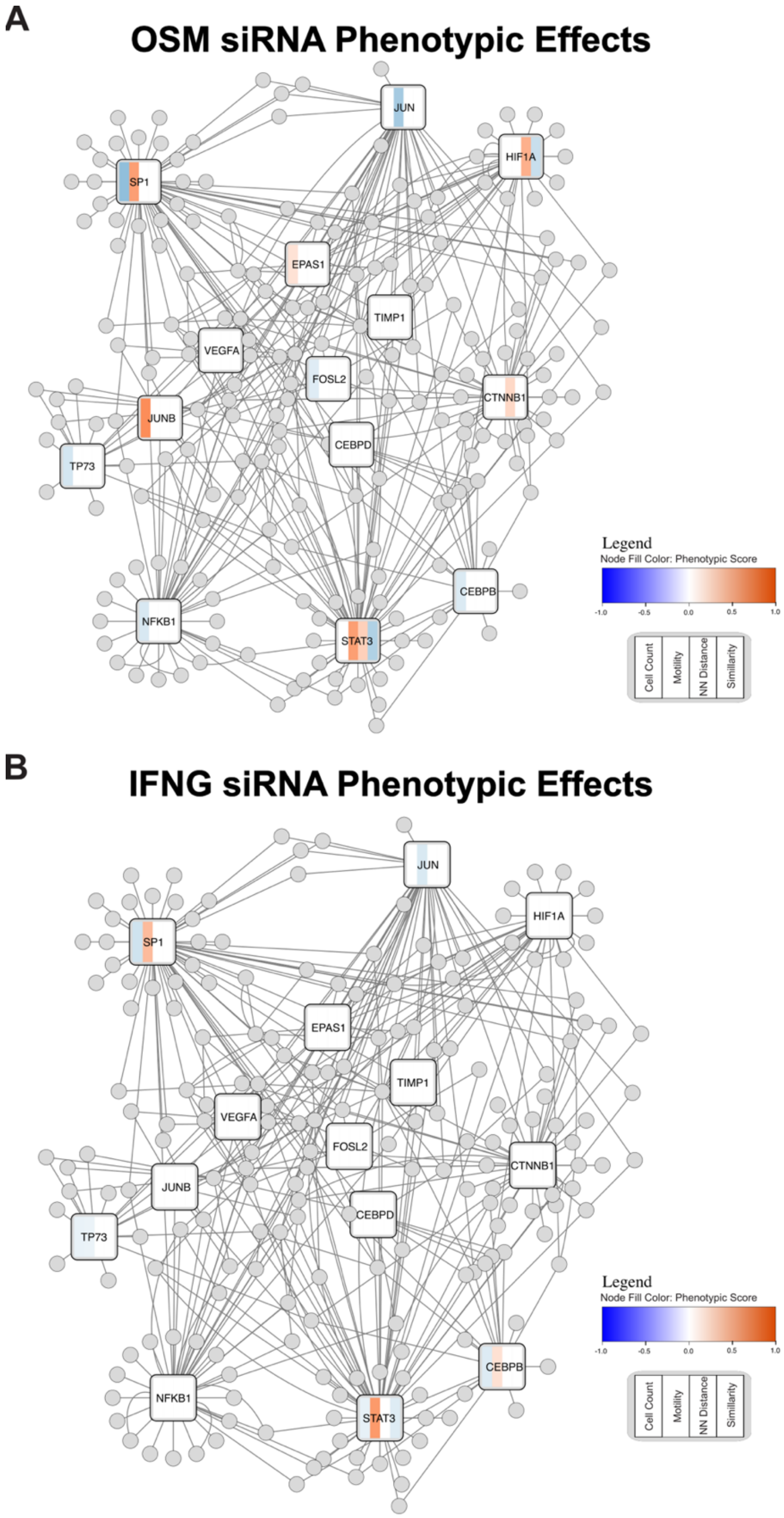

Supplementary Figure 4: Overview of phenotypic effects of siRNA knockdown

(A-B) Integrated molecular network of OSM treated cells. Nodes nominated for siRNA screening are labelled by the phenotypic effect of knockdown in the OSM condition (A) and IFNG condition (B) as compared to scramble control. Non-significant changes in phenotype are colored white.

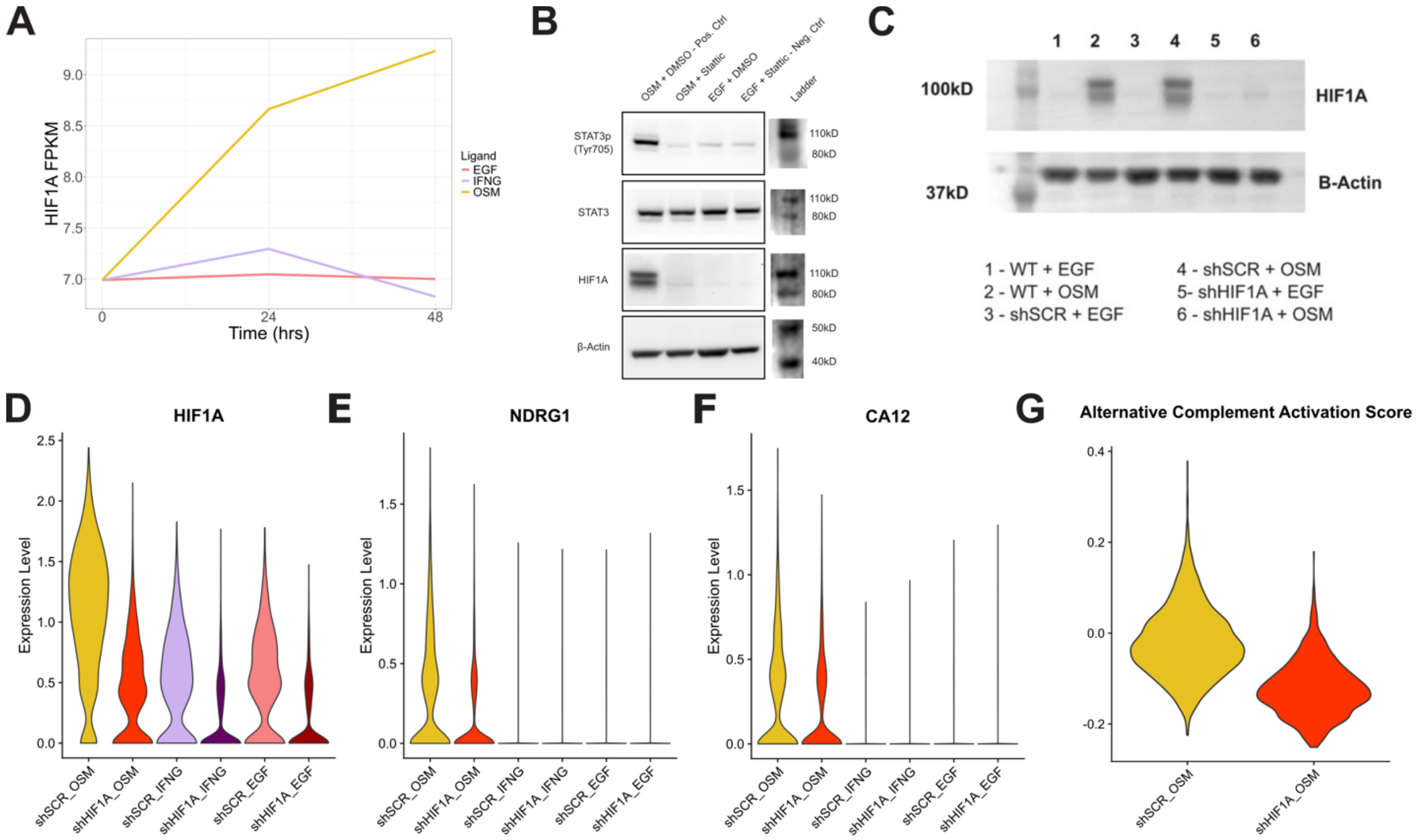

Supplementary Figure 5: scRNAseq supplemental figures

(A) Bulk RNAseq demonstrates that OSM uniquely upregulates HIF1A transcriptionally (n=3). (B) Treatment with a STAT3 inhibitor (Stattic) reduces STAT3 phosphorylation and HIF1A expression in OSM treated cells. Ladder image was processed independently from blot images; contrast and sharpness were enhanced to improve band visibility. (C) Western blot confirming HIF1A knockdown in shHIF1A cell line. The shSCR + OSM condtion serves as a negative control, while WT + OSM serves as the positive. (D) HIF1A expression is significantly reduced in shHIF1A cell lines compared to shSCR. (E-F) Relative gene expression of NDRG1 and CA12: genes that are canonically activated by HIF1A. (G) Knockdown of HIF1A reduces alternative complement activation gene set module score.

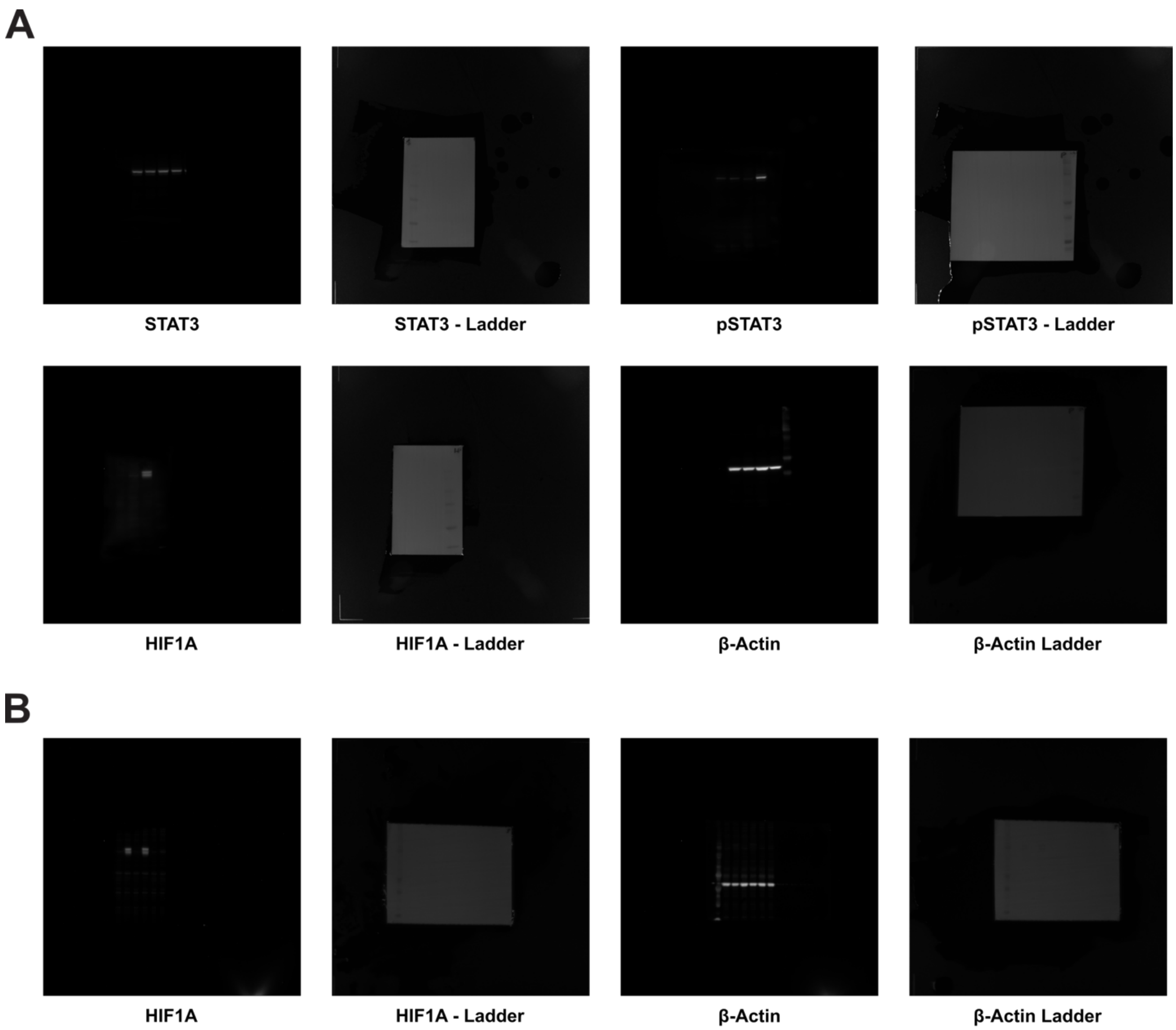

Supplementary Figure 6: Western blot images

(A-B) Uncropped, unadjusted western blot images corresponding to the blots shown in Supplementary Figure 5B and 5C.

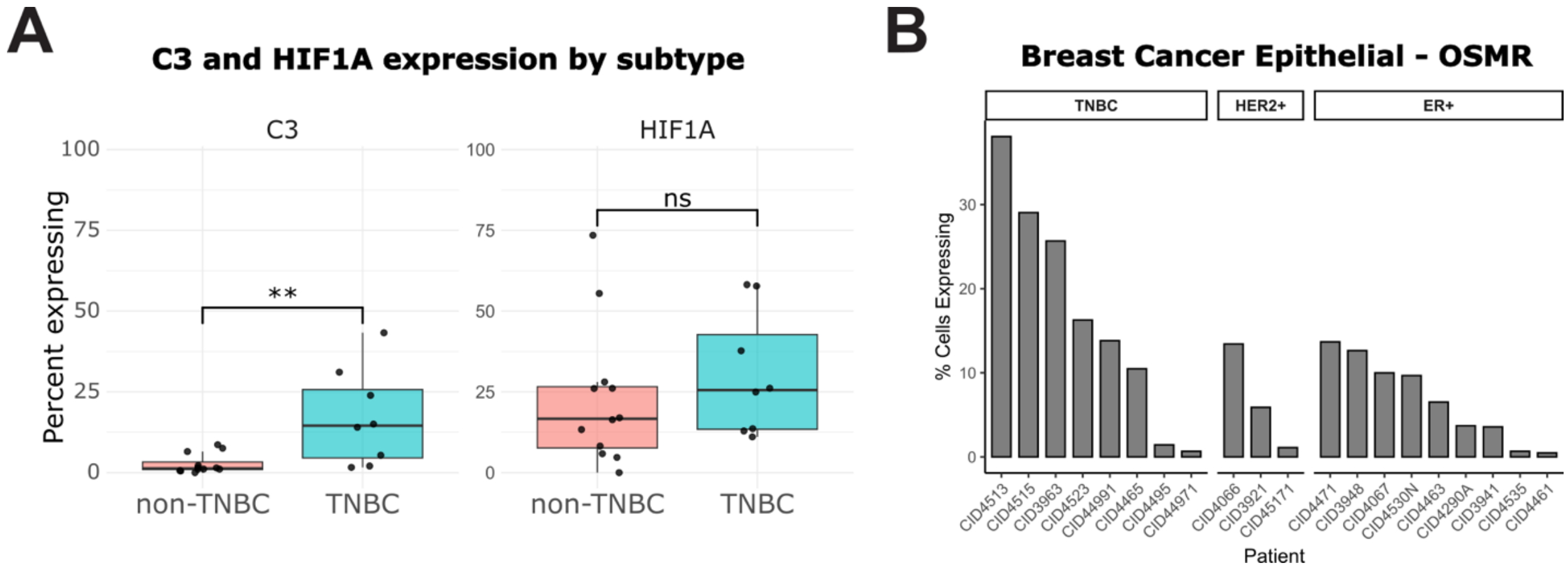

Supplementary Figure 7: The OSM–HIF1A signaling axis is active in basal-like breast cancer and associated with poor prognosis

(A) Boxplot showing the percentage of epithelial cells expressing HIF1A and C3 in individual patients. Significance is shown as follows: (* p-value < .05, ** p-value < .01). (B) Barplot showing the percentage of epithelial cells expressing OSMR for each individual patient.

Name	Vendor	Type	Catalog Number
ON-TARGETplus Non-targeting Pool	Horizon Discovery	ON-TARGETplus SMARTpool - Human	D-001810-10
SP1 ON-TARGETplus SMARTpool - Human	Horizon Discovery	ON-TARGETplus SMARTpool - Human	L-026959-00
STAT3 ON-TARGETplus SMARTpool - Human	Horizon Discovery	ON-TARGETplus SMARTpool - Human	L-003544-00
HIF1A ON-TARGETplus SMARTpool - Human	Horizon Discovery	ON-TARGETplus SMARTpool - Human	L-004018-00
NFKB1 ON-TARGETplus SMARTpool - Human	Horizon Discovery	ON-TARGETplus SMARTpool - Human	L-003520-00
JUN ON-TARGETplus SMARTpool - Human	Horizon Discovery	ON-TARGETplus SMARTpool - Human	L-003268-00
CEBPB ON-TARGETplus SMARTpool - Human	Horizon Discovery	ON-TARGETplus SMARTpool - Human	L-006423-00
EPAS1 ON-TARGETplus SMARTpool - Human	Horizon Discovery	ON-TARGETplus SMARTpool - Human	L-004814-00
JUNB ON-TARGETplus SMARTpool - Human	Horizon Discovery	ON-TARGETplus SMARTpool - Human	L-003269-00
FOSL2 ON-TARGETplus SMARTpool - Human	Horizon Discovery	ON-TARGETplus SMARTpool - Human	L-004110-00
TP73 ON-TARGETplus SMARTpool - Human	Horizon Discovery	ON-TARGETplus SMARTpool - Human	L-003331-00
CEBPD ON-TARGETplus SMARTpool - Human	Horizon Discovery	ON-TARGETplus SMARTpool - Human	L-010453-00
CTNNB1 ON-TARGETplus SMARTpool - Human	Horizon Discovery	ON-TARGETplus SMARTpool - Human	L-003482-00
TIMP1 ON-TARGETplus SMARTpool - Human	Horizon Discovery	ON-TARGETplus SMARTpool - Human	L-011792-00
VEGFA ON-TARGETplus SMARTpool - Human	Horizon Discovery	ON-TARGETplus SMARTpool - Human	L-003550-00
PLK1 ON-TARGETplus SMARTpool - Human	Horizon Discovery	ON-TARGETplus SMARTpool - Human	L-003290-00

Supplementary Table 1: siRNA catalogue

Catalog names and numbers for all siRNA pools utilized in the manuscript.

Supplementary Files

Supplementary Movie 1 – Live cell imaging of MCF10A cells treated with OSM + DMSO vehicle.

Supplementary Movie 2 –Live cell imaging of MCF10A cells treated with OSM + 50 µM Compstatin.
